# Peritoneal Protein Clearance Is a Function of Local Inflammation and Membrane Area Whereas Systemic Inflammation and Comorbidity Predict Survival of Incident Peritoneal Dialysis Patients

**DOI:** 10.3389/fphys.2019.00105

**Published:** 2019-02-18

**Authors:** Zanzhe Yu, Mark Lambie, James Chess, Andrew Williams, Jun-Young Do, Nicholas Topley, Simon J. Davies

**Affiliations:** ^1^Renji Hospital, Shanghai Jiao Tong University School of Medicine, Shanghai, China; ^2^Institute of Applied Clinical Science, Keele University, Stoke-on-Trent, United Kingdom; ^3^Renal Unit, Morriston Hospital, Abertawe and Bro Morgannwg University Health Board, Swansea, United Kingdom; ^4^Division of Nephrology, Yeungnam University Hospital, Daegu, South Korea; ^5^Wales Kidney Research Unit, Division of Infection and Immunity, School of Medicine, Cardiff University, Cardiff, United Kingdom

**Keywords:** large pore flux, survival, mortality, hypoalbuminaemia, interleukin-6, peritoneal solute transport rate, peritoneal membrane, inflammation

## Abstract

It is not clear whether the association of increased peritoneal protein clearance (PPCl) with worse survival on peritoneal dialysis (PD) is a consequence of either local or systemic inflammation or indicative of generalized endothelial dysfunction associated with comorbidity. To investigate this we determined the relationship of PPCl to comorbidity, membrane area (equivalent to low molecular weight peritoneal solute transport rate), local and systemic inflammation and hypoalbuminaemia, and for each of these with patient survival. 257 incident patients from three GLOBAL Fluid Study centers were included in this analysis. Clinical profiles were collected at baseline along with a peritoneal equilibration test, 24-h dialysate protein and paired plasma and dialysate cytokine measurements. Although peritoneal protein clearance was associated with increased age and severe comorbidity on univariate analysis, only dialysate IL-6, peritoneal solute transport rate, plasma albumin and cardiac comorbidities (ischaemic heart disease and left ventricular dysfunction) were independent explanatory variables on multivariate analysis. While peritoneal protein clearance and daily peritoneal protein loss were associated with survival in univariate analysis, on multivariate analysis only plasma IL-6, age, residual kidney function, comorbidity, and plasma albumin were independent predictors. Peritoneal protein clearance is primarily a function of peritoneal membrane area and local membrane inflammation. The association with comorbidity and survival is predominantly explained by its inverse relationship to hypoalbuminaemia, especially in diabetics.

## Introduction

**FIGURE 1:**
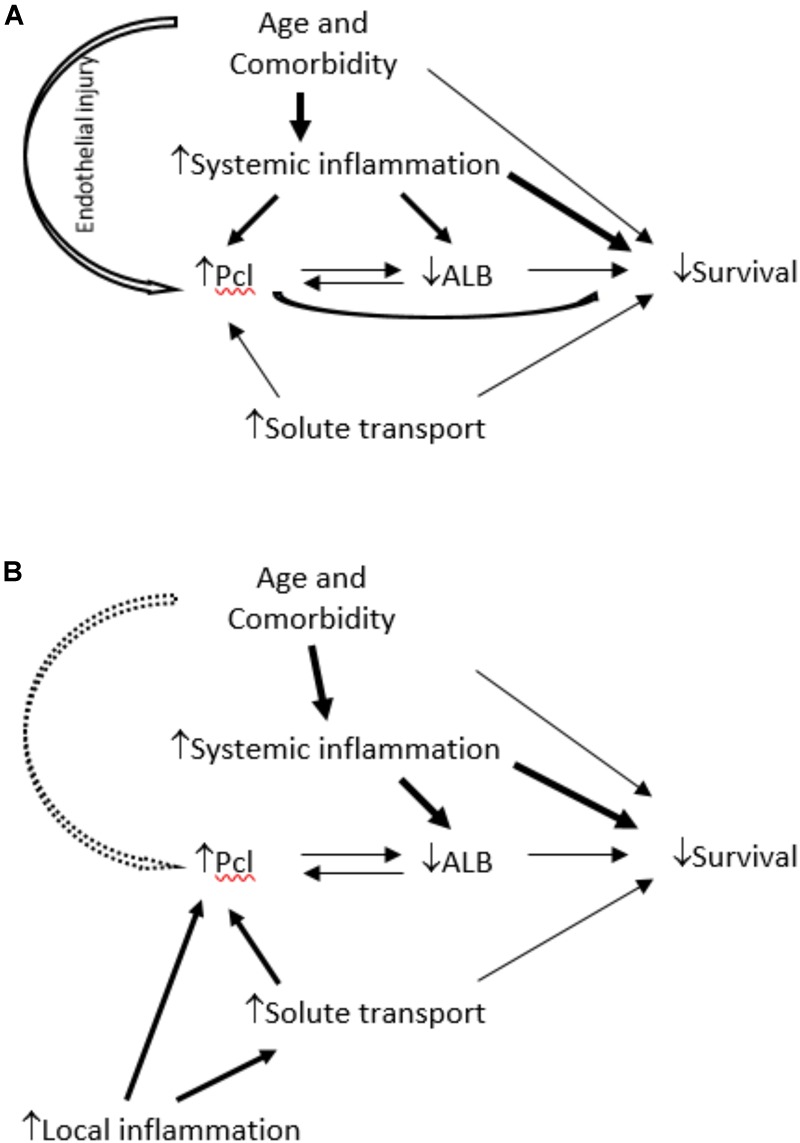
In **(A)**, the link between comorbidity and PPCl is considered to be a consequence of three possible mechanisms: increased systemic inflammation, systemic endothelial dysfunction or its association with albumin – considered as potentially bidirectional because although increased protein clearance will cause greater protein losses there is also mathematical coupling. The model proposed in **(B)** light of these study findings, is that local not systemic inflammation is the predominant determinant of PPCl and an independent link with comorbidity, if present, is weak. The link between PPCl and survival is subordinate to that with plasma albumin, but the bidirectional association is not fully resolved.

Peritoneal protein clearance (PPCl) has been shown to relate to comorbidity in several studies (Heaf et al., 2005; Szeto et al., 2005; Johansson and Haraldsson, 2006; Van Biesen et al., 2006; Perl et al., 2009; Sánchez-Villanueva et al., 2009; Balafa et al., 2011) and in many cases to worse survival on peritoneal dialysis (PD). An attractive explanatory hypothesis would be that increased PPCl, due to the increased flow of proteins through the large pore pathway of the peritoneal microvasculature might reflect systemic endothelial barrier dysfunction and thus be a biomarker of vascular comorbidity and worse survival (Figure 1A). However, data from the Netherlands group (Balafa et al., 2011) demonstrated that while baseline peritoneal albumin and protein clearances from a 4 h dwell with 3.86% glucose dialysate were associated with signs of comorbidity there was no measurable effect on patient survival. Thus the potential relationships between PPCl, comorbidity and survival remains to be fully determined, and to date the impact of local peritoneal inflammation has not been studied.

According to the three pore model, PPCl should be proportional to peritoneal solute transport rate (PSTR), given that both large and small pores are located within peritoneal capillaries. Local peritoneal but not systemic inflammation is the main predictor of PSTR at the commencement of dialysis, (Lambie et al., 2013) reflecting small pore area, but could additionally increase protein losses by causing a relative increase in large pore area. The purpose of this analysis was to distinguish between local versus systemic determinants of PPCl and determine how these relate to survival using a subgroup of centers in the Global Fluid Study in which peritoneal protein losses were measured.

## Materials and Methods

This was a subgroup analysis of the GLOBAL Fluid study cohort. A detailed description of the GLOBAL Fluid Study has been published previously (Lambie et al., 2013). In brief, it is an international, multi-center, prospective, observational cohort study where 10 centers from the United Kingdom, Canada, and South Korea sought to enroll all PD patients starting or already on PD between 2002 and 2008, with the only exclusion criterion being lack of informed consent and <18 years of age. For this analysis, three of the ten centers in the GLOBAL Fluid study were included, two in the United Kingdom, one in South Korea, as these centers routinely measured daily dialysate protein losses. All the incident patients in these three centers in the GLOBAL Fluid study during 2002 to 2008 were included. Ethical approval was obtained from the Multi-Center Research Ethics Committee for Wales covering the United Kingdom, and the Kyungpook National University Hospital Ethics Committee for the Korean center. Written informed consent was obtained from all subjects and they were followed until their death or the censor date, December, 2011. Two patients using an amino acid based solution were excluded from the analysis as the effects of this solution on local inflammatory cytokine production are unknown.

### Prospective Collection of Routine Clinical Measurements

Samples of dialysate and plasma and extended clinical phenotype were collected prospectively when patients were undergoing routine planned clinical assessments of membrane function within three months of treatment start. The clinical characteristics, including membrane function, PPCl, biochemistry profiles, and comorbidity were estimated locally. Both of the two United Kingdom centers used the original 2.27% PET for the measurement of PSTR (D/P creatinine) and the South Korea center calculated the PSTR based on 4 h result from a 3.86% exchange.

Comorbidity was documented according to the externally validated Davies comorbidity score (Davies et al., 2002). Briefly, 7 comorbid domains were considered, including non-cutaneous malignancy, ischemic heart disease (IHD), peripheral vascular disease (PVD) (including cerebrovascular and renovascular disease), left ventricular dysfunction (LVD), diabetes mellitus (DM), systemic collagen vascular disease, and ‘other’ (any other condition known to reduce life expectancy in the general population). The comorbidity score for each patient was defined as the number of these domains affected. The comorbidity grade was then derived from the comorbidity score. Grade 0 (low risk) was a zero score, grade 1 (medium risk) was a score of 1–2, and grade 2 (high risk) a cumulative score of ≥3.

The peritoneal dialysate protein loss was measured from the collection of 24-h peritoneal dialysate effluent. A validated correction factor was used to calculate PPCl: 24 h dialysate protein loss/(serum albumin/0.4783), (Haraldsson, 1995) and expressed as mL/day.

### Sample Analysis

Dialysate and plasma samples were stored locally at -80°C, and were sent to a central laboratory for measurements of IL-1β, TNF-α, IL-6 and INF-γ by electrochemiluminescence immune assay using the commercially available Pro-Inflammatory I 4-plex (Meso-Scale Discovery, Gaithersburg, MD, United States). For correlations and comparisons, dialysate appearance rate (AR) of cytokines was calculated from the dialysate concentration divided by dwell time. The median intra-assay coefficient of variation was 8.0.

### Statistical Analysis

Continuous data were expressed as mean values ± SD for normally distributed variables; otherwise median (± inter-quartile range) was used unless they could be log_10_ transformed. One-way ANOVA or Student’s *t*-test were used to examine differences in normally distributed continuous data or baseline categorical variables, while Mann-Whitney or Kruskal-Wallis tests were used for non-parametric variables. The relationship between PPCl and continuous variables was examined by Pearson correlation coefficient.

Mixed linear modeling was used to identify the determinants of PPCl with a random intercept for center to account for the observed center effects. Kaplan-Meier plots with log rank tests and Cox regression with robust standard errors were used for survival analysis. Collinearity was assessed using a variation inflation factor of >5 as a pree-specified cutoff to indicate a problem.

Significance was pre-specified for *p*-values <0.05 and 95% confidence intervals not crossing the value for no effect. All statistical analyses were performed using SPSS 20 (SPSS Inc., Chicago Ill., United States) apart from the mixed linear model, which was performed by MLwin software (Version 2.22, Center for multilevel modeling University of Bristol).

## Results

### Patient and Membrane Characteristics

A total of 257 patients in the three centers were included in the study. Table 1 displays the main baseline characteristics. There were significant differences between centers in a variety of variables, including BMI, comorbidity, PSTR, residual renal function and biochemical characteristics and all the plasma and dialysate cytokines levels. The dialysis regime was also different among centers.

**Table 1:** Patient demographic and biochemical characteristics, peritoneal membrane function and systemic and dialysate inflammatory cytokines by center.

Center	G05 (n = 77)	G01 (n = 57)	K03 (n = 123)
Age (yr)	56.3 ± 15.3	57.1 ± 14.3	53.3 ± 14.6
Gender (M/F)	47/30	37/20	72/51
BMI (kg/m^2^)	27.0 ± 5.4	28.2 ± 4.5	23.4 ± 2.9
**Comorbidity Grade n (%)**			
Low	23 (29.9%)	26 (45.6%)	49(40.0%)
Medium	43 (55.8%)	27 (47.4%)	73 (59.2%)
High	11 (14.3%)	4 (7.0%)	1 (0.8%)
DM (yes/no)	27/50	14/43	59/64
Day of PET (day)	42 ± 20	25 ± 19	38 ± 14
Alb (g/L)	35.8 ± 4.2	37.6 ± 4.7	33.4 ± 5.1
Hgb (g/L)	11.9 ± 1.5	11.4 ± 1.4	8.7 ± 2.4
Urine volume (ml)	1203 ± 785	1324 ± 813	1027 ± 620
PSTR	0.77 ± 0.14	0.60 ± 0.12	0.73 ± 0.10
CAPD/APD	59/18	57/0	123/0
Icodextrin (with/without)	20/57	0/57	16/107
Bicarbonate buffered solution (with/without)	2/75	28/29	21/102
PPCl (ml/day)	89.9 ± 33.6	89.7 ± 46.2	95.5 ± 48.3
Dialysate IL-1β AR (pg/min)	0 (0–0)	0 (0–2.12)	0 (0–0.32)
Dialysate TNF-α AR (pg/min)	0 (0–1.78)	3.30 (1.15–8.64)	1.69 (0.28–5.62)
Dialysate IL-6 AR (pg/min)	32.9 (8.8–59.6)	41.7 (23.1–90.6)	73.9 (30.7–135.0)
Dialysate IFN-γ AR (pg/min)	10.5 (0–46.4)	0 (0–15.2)	0 (0–3.3)
Plasma IL-1β (pg/ml)	0.12 (0.06–0.26)	0.01 (0–0.07)	0.05 (0–0.21)
Plasma TNF-α (pg/ml)	7.2 (5.7–8.7)	8.3 (6.7–9.7)	17.6 (15.4–22.0)
Plasma IL-6 (pg/ml)	1.5 (0.7–2.8)	0.8 (0.2–2.3)	2.0 (1.2–3.7)
Plasma IFN-γ (pg/ml)	1.0 (0.4–1.6)	0.7 (0–2.2)	2.2 (1.3–4.0)

*BMI, body mass index; PSTR, Peritoneal Solute Transport Rate expressed as dialysate/plasma creatinine at 4 h; PCl, protein clearance; Comorbidity Grade – Davies Comorbidity grade as defined previously(Davies et al., 2002); AR, appearance rate.*

### Univariate Correlation to PPCl

**FIGURE 2:**
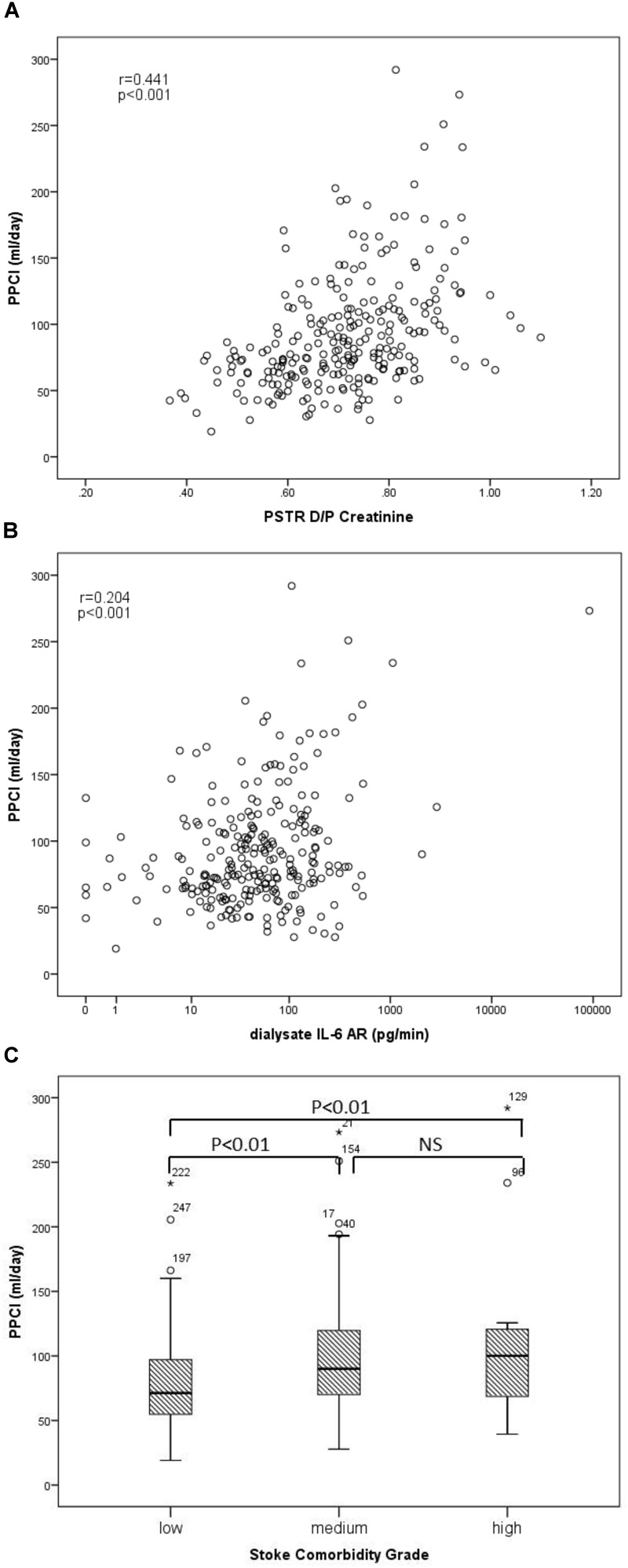
Univariable associations of peritoneal protein clearance. **(A)** PPCl and PSTR (D/P creatinine), **(B)** PPCl and Dialysate IL-6 AR (pg/min), (r-correlation coefficient of univariable assosication), and **(C)** PPCl and comorbidity grade.

The univariate correlations between PPCl, patient and membrane characteristics, as well as dialysate and plasma inflammatory cytokines are presented in Table 2. PPCl was positively related to age and PSTR (Figure 2A). A strong negative correlation was seen between PPCl and serum albumin, which is in part a function of mathematical coupling. No significant difference in PPCl was found according to icodextrin use, biocompatible solution use or modality (CAPD versus APD).

**Table 2A:** Univariate associations between PPCl, patient and membrane characteristics, plasma and dialysate inflammatory cytokines.

	Correlation Coefficient^a^
Age	0.15
BMI	0.05
Serum albumin	-0.47
PSTR	0.44
24 h urine volume	-0.02
Dialysate IL-1β AR	0.04
Dialysate TNF-α AR	0.14
Dialysate IL-6 AR	0.20
Dialysate IFN-γ AR	0.04
Plasma IL-1β	0.02
Plasma TNF-α	0.06
Plasma IL-6	0.13
Plasma IFN-γ	-0.01

*a, Pearson correlation coefficient for normal distributed variables and Spearman correlation coefficient for non-normal distributed variables. BMI, body mass index; PP, pulse pressure; PSTR, Peritoneal Solute Transport Rate, (4 h dialysate/plasma creatinine); Ccr, creatinine clearance; IL, interleukin; TNF, tumor necrosis factor; INF, interferon; AR, appearance rate.*

Both dialysate IL-6 appearance rate (AR) and less strongly plasma IL-6 were positively correlated to PPCl (Figure 2B and Table 2) Dialysate but not plasma TNF-α were related to PPCl. PPCl was associated both with the overall severity of comorbidity, (Figure 2C) and with specific comorbidities, namely IHD, DM, and LVD (Table 2B).

**Table 2B:** Univariate associations between PPCl and patient characteristics.

		PPCl, ml/day mean ± SD
	
Gender	Male	96.3 ± 45.5
	Female	87.4 ± 41.1
Comorbidity grade	Low	79.4 ± 37.0
	Medium	99.6 ± 43.1
	High	109.7 ± 67.0
PD modality	CAPD	92.4 ± 44.6
	APD	94.5 ± 33.9
Icodextrin	Without	90.2 ± 35.5
	With	92.9 ± 45.1
Biocompatible solution	Without	88.1 ± 38.5
	With	93.6 ± 45.1
DM	Without	85.8 ± 39.59
	With	103.1 ± 48.12
IHD	Without	88.46 ± 39.93
	With	112.24 ± 55.72
LVD	Without	89.98 ± 40.34
	With	118.53 ± 66.01
Malignancy	Without	92.15 ± 43.8
	With	106.14 ± 46.83
PVD	Without	91.38 ± 43.86
	With	101.61 ± 43.43
Collagen disease	Without	92.69 ± 43.86
	With	78.86 ± 48.93

*DM, Diabetes Mellitus; IHD, Ischaemic Heart Disease; LVD, Left Ventricular Dysfunction; PVD, Peripheral Vasculare Disease.*

### Multivariate Model for PPCl

To account for clustering by center, a random intercept term was used for center. Gender, age, comorbidity grade, Log_10_ dialysate IL-6 AR, Log_10_ plasma IL-6, and PSTR were included in the multivariate model.

With PPCl as the dependent variable, dialysate IL-6 AR, plasma albumin and PSTR were the independent explanatory variables (Table 3). Further models substituting specific comorbid domains instead of the overall comorbidity grade showed that IHD and LV dysfunction predicted higher PPCl, but not DM, PVD or other comorbidities (Supplementary Tables 1, 2). A sensitivity analysis using daily peritoneal protein loss as an alternative measure to peritoneal protein clearance showed a similar pattern but no correlation to plasma albumin, (Supplementary Table 4). Tests for collinearity demonstrated no significant effect, with the highest variance inflation factor of 1.49 for albumin. Variables included in models were selected *a priori*, i.e., on the basis of previous reported associations or biological plausibility, with no statistical criteria used for selection.

**Table 3:** Multivariable model for PPCl^a^.

	β	95% CI	*P*-value
PSTR (for each 0.1 increase D/P _creatinine_)	11.88	7.83–15.93	<0.001
Dialysate IL6 AR (for each 10 fold increase)	8.70	0.82–16.59	0.03
Plasma IL6 (for each 10 fold increase)	5.55	-10.05–21.15	0.49
Albumin (for each 1g/L increase)	-2.70	-3.76–1.63	<0.001
Age (year)	0.04	-0.28–0.35	0.82
Male gender	-0.45	-9.46–8.56	0.92
Comorbidity Grade 1 (compared with Grade 0)	6.72	-3.05–16.49	0.44
Comorbidity Grade 2 (compared with Grade 0)	10.01	-9.81–29.83	

*a, mixed linear model with random intercept for center. PPCl, peritoneal protein clearance; PSTR, peritoneal solute transport rate; AR, appearance rate; IL-6, interleukin-6.*

### Survival Analysis

**FIGURE 3:**
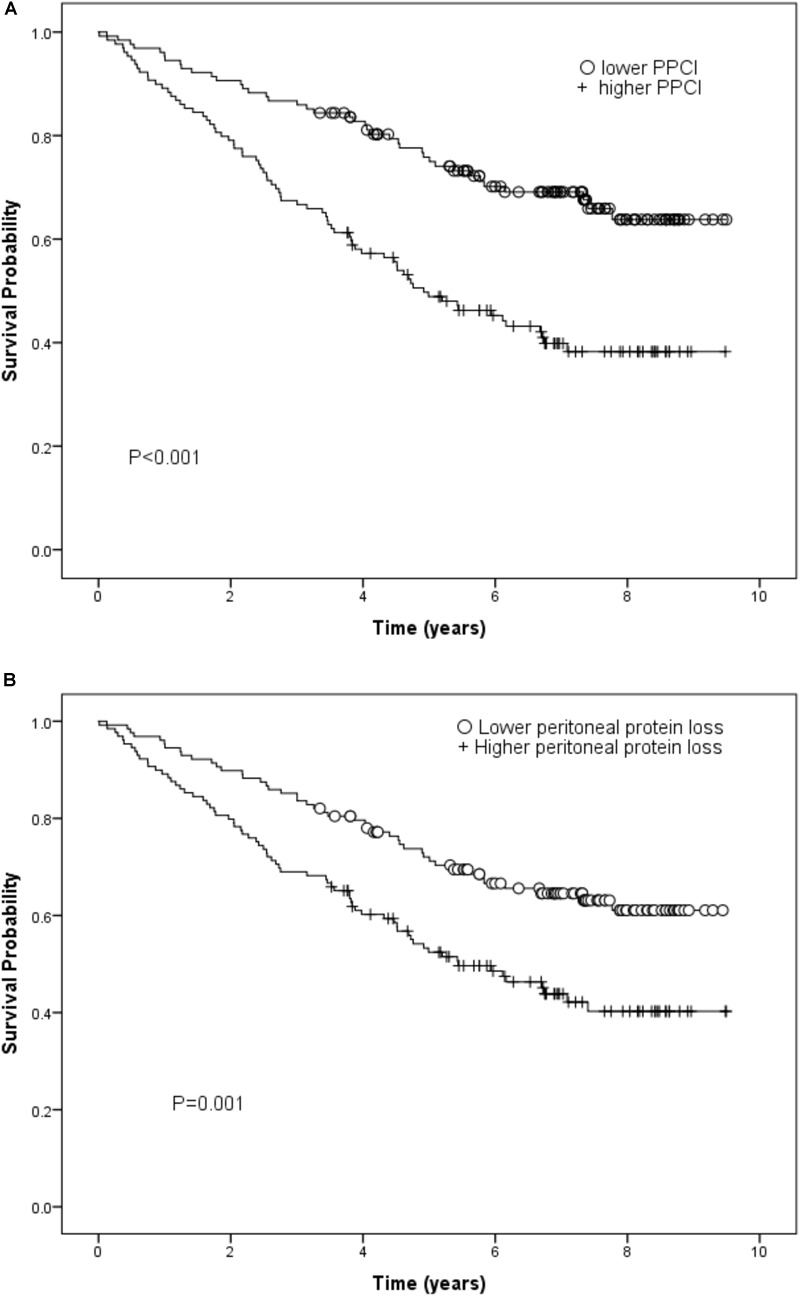
**(A)** Kaplan-Meier curve for peritoneal protein clearance (PCl) (log rank test) and **(B)** Kaplan-Meier curve for daily peritoneal protein loss (log rank test).

There were 115 deaths in the 257 patients during a median follow up of 37 months. In the Kaplan-Meier plot, the survival rate was compared between patients with PPCl split by the median value of the whole group. Higher PPCl was shown to be related to worse overall outcome and this was also true for daily peritoneal protein loss (Figure 3).

Using Cox regression, in the basic model which also included gender, renal Kt/V, local and systemic inflammation, the independent predictors of survival were age, renal Kt/V, systemic IL-6, PSTR, and comorbidity. On adding PPCl into the model, the predictive value of PSTR was displaced by a significant association with PPCl. By further adding plasma albumin into the model, the significant prediction of mortality by PPCl was displaced, with age, renal Kt/V, plasma IL-6, comorbidity grade, and plasma albumin the independent predictors for survival (Table 4). Application of the likelihood ratio test confirmed that addition of PPCl to the models confirmed that it was only a significant predictor until plasma albumin, with which it is correlated was added to the model, resulting in an overall better prediction. The lack of association of PPCl with survival was not altered by substituting specific comorbidities into the model (see Supplementary Table 3). When daily protein loss was substituted for PPCl in the third model this was not a significant predictor (Hazard Ratio 1.06/g/day, (95%CI 0.96–1.17). A sensitivity analysis including hemoglobin as a covariate made no substantive difference.

**Table 4:** Cox regression models of patient survival stratified by center – with PPCl and plasma albumin as additional covariates.

	Base Model			Base Model + PPCl			Base Model + PPCl + Alb		
	HR	95%CI	*P*-value	HR	95%CI	*P*-value	HR	95%CI	*P*-value
Age (per year)	1.075	1.05–1.1	<0.01	1.076	1.05–1.1	<0.01	1.077	1.05–1.1	<0.01
Gender (Female)	1.154	0.75–1.77	0.51	1.179	0.76–1.83	0.47	1.192	0.77–1.85	0.43
PSTR	9.912	1.8–54.44	<0.01	3.393	0.42–27.26	0.25	2.681	0.36–20	0.34
Plasma IL-6 (per 10 fold increase)	2.397	1.18–4.89	<0.05	2.239	1.06–4.71	<0.05	2.201	1.08–4.47	<0.05
Comorbidity grade 1	2.271	1.4–3.69	<0.01	2.063	1.26–3.38	<0.01	1.827	1.09–3.05	<0.05
Comorbidity grade 2	7.787	3.36–18.04	<0.01	7.198	3.22–16.1	<0.01	4.933	2.07–11.78	<0.01
Peritoneal IL-6 AR (per log order)	1.098	0.77–1.56	0.6	1.047	0.72–1.53	0.81	1.040	0.7–1.54	0.84
Renal Kt/V (per unit)	0.614	0.42–0.9	<0.05	0.634	0.43–0.93	<0.05	0.658	0.46–0.95	<0.05
PfopPCl (per ml/min)				1.005	1–1.01	<0.05	1.002	1–1.01	0.5
Plasma Albumin (per g/l)							0.924	0.87–0.98	<0.01

*PPCl, peritoneal protein clearance; PSTR, peritoneal solute transport rate; AR, appearance rate; IL-6, interleukin-6; HR, hazard ratio, CI, confident interval.*

## Discussion

The purpose of this sub-analysis of the GLOBAL Fluid study was to determine whether PPCl is a consequence of local peritoneal membrane inflammation or a reflection of vascular injury associated with systemic inflammation and comorbidity and, in turn understand how these associate with subsequent patient survival. This is the first time to our knowledge that both local and systemic inflammation, PSTR, PPCl, and comorbid conditions have all been measured at the same time. We demonstrated that PPCl is a function of local inflammation (as reflected by the product of effective membrane area and local dialysate IL-6 appearance rate) and not systemic inflammation in patients commencing PD. The association of comorbidity with PPCl is predominantly explained by the inverse correlation to plasma albumin. This is for several reasons including the reverse acute phase response associated with systemic inflammation, which is in part causal, as a greater protein loss will cause the albumin to drop, but predominantly due to mathematical coupling. The calculation of PPCl has albumin as its denominator which will be depressed in the presence of systemic inflammation. Thus PPCl is not an independent predictor of survival in this analysis which takes both systemic and local inflammation into account, suggesting that the previously reported associations were not due to an association with systemic inflammation or endothelial barrier dysfunction, as demonstrated in the revised causal diagram, Figure 1B.

Several studies have shown that membrane inflammation is associated with high PSTR, primarily a measure of the effective membrane area in incident patients (van Esch et al., 2004; Hwang et al., 2009; Lambie et al., 2013). However, until now it has not been clear how this relates to the variability in large pore flux. It is well known that the large pore flux (and thus PPCl) increases dramatically during peritonitis, i.e., in the presence of severe inflammation that includes an influx of neutrophils, but this has been less clear in the context of a stable, non-infected peritoneal cavity. A study in 40 prevalent CAPD patients found that the dialysate appearance of IL-6 correlated with albumin and IgG clearances, which was also related to PSTR at the same time (Pecoits-Filho et al., 2002). It was not clear from this study, however, whether the inflammation associated increase of PPCl was fully explained by an increase in membrane area. Our results indicate that local IL-6 production, increases PPCl not just via increased effective area (PSTR), but also through an increase in the density of large pores leading to a more leaky membrane.

Although the hypothesis that PPCl reflects generalized endothelial dysfunction is attractive, our findings from the multivariate analysis did not support this assumption. We demonstrated that comorbidity was not an independent predictor of PPCl (after controlling for local and systemic inflammation, PSTR and plasma albumin). This is consistent with previous findings in a longitudinal single center cohort study, (Yu et al., 2014) where no significant correlation between PPCl and comorbidity was observed, albeit in a highly selected cohort (restricted to patients on PD for more than 4 years). This longitudinal study also found that PPCl decreases with time for a given membrane area, despite the fact that comorbidity tends to accumulate with time on PD, suggesting that progressive membrane fibrosis is occuring (Yu et al., 2014). Furthermore, detailed analysis of the transcapillary escape rate of albumin, a measure of systemic endothelial barrier dysfunction, found that this was highly abnormal in PD patients, in keeping with an impaired glycocalyx in dialysis patients, (Vlahu et al., 2012) but found no relationship to either systemic inflammation or PPCl. Given that this was a study of prevalent patients who may have acquired membrane fibrosis its interpretation requires caution, but it is nevertheless further evidence that systemic impairment of endothelial barrier function is not a major determinant of PPCl (Yu et al., 2012). PPCl is therefore predominantly a function of peritoneal membrane inflammation and membrane surface area rather than a reflection of systemic endothelial injury.

In addition to the analyses testing our primary hypothesis, we undertook exploratory analyses of the association between different comorbidities and PPCl. Whereas DM was not independently associated with PPCl, this was the case for IHD and LV dysfunction (Supplementary Table 2). The explanation for this is not certain, but we did observe that patients with IHD had greater systemic inflammation [plasma IL-6 IHD 2.40 pg/ml (1.30–4.35) vs. DM 1.86 pg/ml (1.03–3.02)], whereas those with DM had a more marked reduction in plasma albumin. This agrees with the findings of the previously cited single center study, (Yu et al., 2012) incorporating a much wider biomarker profile, which found systemic inflammation was more pronounced in IHD whereas platelet activation and systemic albumin leak (not peritoneal) was more significant in DM, suggesting differential effects according to type of comorbidity on endothelial function. Other factors to be taken into account include the different kinetics of peritoneal protein loss as compared to systemic protein leak on plasma albumin (which can be recycled) and other factors that could theoretically increase peritoneal protein losses such as transcapillary hydrostatic pressure. One of the limitations of the present study is that it may not have been large enough to demonstrate different effects by comorbidity on PPCl and survival.

There were a number of other limitations of our study. Firstly, athough a validated correction factor was used to estimate total protein from serum albumin a direct measurement of total plasma protein may have given different results. It is possible that in extreme systemic inflammation, total serum protein may be underestimated and PPCl overestimated because while albumin is a negative acute-phase protein, other proteins increase in inflammation. Thus, the association between systemic inflammation and PPCl may have been amplified, as it may have been in previous studies. However, this does not alter the conclusions of this study, namely that PPCl is more associated with local than systemic inflammation. Secondly, albumin, the predominant plasma and dialysate protein, is able to pass through small pores (predominantly by convection) as well as large pores (Lindholm et al., 1987). Ideally, a series of proteins at different molecular weights should be measured to precisely dissect out the contribution of the different pore sizes, a procedure that is logistically challenging for a large prospective epidemiological study such as this. Thirdly, proteinuria, another route of protein loss that can affect plasma albumin levels in PD patients, (Gama-Axelsson et al., 2012) would be measured as this may differ by comorbidity. Unfortunately, these measures were not available in this study. Fourthly, a larger study may have been able to show independent effects of more of the covariates on survival; for example a recent study (Mehrotra et al., 2015) of more than 10,000 new patients found a significant association between high solute transport and survival that was not observed here.

Finally, as for all observational studies, we cannot prove causality in the association of peritoneal inflammation with PPCl, although it fits a lot of the Bradford-Hill criteria for causality. Our survival analysis is primarily disproving a causal association suggested by previous studies so this caveat is less relevant. An interventional trial of an agent that turns off intraperitoneal inflammation would be required to investigate causality. The strengths of the present study include the multi-center multinational nature of the study whilst still allowing for clustering by center in the analysis, the large number of patients studied, length of follow up and adjustment for multiple confounding covariates.

### Summary

In conclusion, we have shown that PPCl is predominantly determined by peritoneal membrane area and local inflammation. The predictive value of PPCl on survival is mainly through its coupling to hypoalbuminaemia rather than a direct reflection of endothelial injury. PPCl is not a good marker for systemic endothelial function but a reflection of local peritoneal membrane inflammation.

## Author Contributions

ZY undertook the primary analysis of the data and wrote the main draft of the paper. ML supervised the data analysis. JC and AW recruited the patents from Swansea (United Kingdom center). JC assisted with database development. J-YD recruited patients from the Korean center. NT and SD co-lead the Global Fluid Study. SD recruited patients from the Stoke-on-Trent Center.

## Conflict of Interest Statement

ZY, ML, and SD receive research funding from Baxter Healthcare (Clinical Evidence Council). The remaining authors declare that the research was conducted in the absence of any commercial or financial relationships that could be construed as a potential conflict of interest.
