# Supplementary material for: Peritoneal Protein Clearance Is a Function of Local Inflammation and Membrane Area Whereas Systemic Inflammation and Comorbidity Predict Survival of Incident Peritoneal Dialysis Patients
Source: Front Physiol. 2019 Feb 18;10:105. doi: 10.3389/fphys.2019.00105 (PMC6387967; doi:10.3389/fphys.2019.00105)
Supplement: Supplementary file 1 [file Data_Sheet_1.PDF]

Supplementary Material: Peritoneal protein clearance is a function of local inflammation and membrane area whereas systemic inflammation and comorbidity predict survival of incident peritoneal dialysis patients

Zanzhe Yu, Mark Lambie, James Chess, Andrew Williams, Jun-Young Do, Nicholas Topley, Simon J Davies.

Tables S1-4

Supplemental Table 1: Plasma albumin (g/L) according to comorbidity status

	Diabetes	IHD	LVD	Malignancy	PVD	Collagen disease
**without**	36.77±4.37	35.36±5.1	35.21±4.9	35.04±5.11	35.28±5.0	35.02±5.0
**n**	157	213	234	250	228	254
**with**	32.37±4.89	33.59±4.6	33.57±5.6	35.71±2.36	33.31±4.9	38±3.4
**n**	100	44	23	7	29	3
**p value[a]**	<0.001	<0.05	0.14	0.5	<0.05	0.31

a, unpaired t test

DM, diabetes; IHD, ischemic heart disease; PVD, peripheral vascular disease; LVD, left ventricular dysfunction

Supplemental table 2: multivariate model for PPCl – regarding different comorbidity domain

	Diabetes Model			IHD Model			LVD Model			PVD Model		
	**β**	**95% CI**	**P value**	**β**	**95% CI**	**P value**	**β**	**95% CI**	**P value**	**β**	**95% CI**	**P value**
**PSTR (for each 0.1 increase)**	11.55	7.51-15.593	<0.001	11.64	7.651-15.63	<0.001	11.54	7.556-15.517	<0.001	11.44	7.413-15.47	<0.001
**Dialysate IL6 AR (per log order)**	9.60	1.8-17.41	0.02	8.39	0.6-16.17	0.04	8.74	0.99-16.48	0.03	9.55	1.74-17.35	0.02
**Plasma IL6 (per log order)**	-2.12	-6.32-2.08	0.32	-1.71	-5.86-2.45	0.42	-2.07	-6.21-2.07	NS	-2.02	-6.21-2.17	0.35
**Albumin (per g/L increase)**	-3.18	-4.23--2.14	<0.001	-2.95	-3.92--1.97	<0.001	-2.98	-3.95--2.01	<0.001	-3.08	-4.07--2.09	<0.001
**Age (per year)**	0.08	-0.23-0.4	0.60	0.02	-0.3-0.33	0.91	0.07	-0.24-0.37	0.33	0.09	-0.22-0.4	0.55
**Male gender**	0.60	-8.36-9.56	0.89	0.39	-8.48-9.26	0.93	-0.09	-8.97-8.79	0.98	0.70	-8.31-9.71	0.88
**Diabetic**	-2.77	-12.52-6.99	0.58									
**IHD**				13.94	2.16-25.73	0.02						
**LVD**							18.64	3.7-33.58	0.01			
**PVD**										-0.25	-14.4-13.9	0.97

DM, diabetes; IHD, ischemic heart disease; LVD, left ventricular dysfunction; PVD, peripheral vascular disease; PPCl, protein clearance; PSTR, peritoneal solute transport rate; AR, appearance rate; IL-6, interleukin-6

Supplemental Table 3: Cox regression model of patient survival stratified by center- with different comorbidity domain in the model

	diabetes model			IHD model			LVD model			PVD model		
	HR	95%CI	P value	HR	95%CI	P value	HR	95%CI	P value	HR	95%CI	P value
**Age (per year)**	1.09	1.06-1.11	<0.001	1.07	1.05-1.09	<0.001	1.07	1.05-1.1	<0.001	1.07	1.05-1.1	<0.001
**Gender (Female)**	1.15	0.75-1.75	0.53	1.17	0.76-1.79	0.48	1.18	0.76-1.82	0.46	1.11	0.72-1.71	0.63
**PSTR**	1.06	0.13-8.49	0.96	1.65	0.2-13.86	0.65	1.43	0.16-12.86	0.75	1.37	0.17-11.24	0.77
**Plasma IL-6 (per log order)**	3.00	1.57-5.74	<0.001	2.33	1.23-4.42	<0.01	2.50	1.36-4.61	<0.01	2.62	1.42-4.85	<0.01
**Peritoneal IL-6 AR (per log order)**	0.94	0.65-1.36	0.74	1.02	0.69-1.52	0.92	0.98	0.67-1.45	0.93	0.99	0.68-1.46	0.97
**Renal Kt/V (per unit)**	0.68	0.47-0.97	<0.05	0.72	0.5-1.02	0.07	0.72	0.51-1.02	0.06	0.75	0.53-1.06	0.11
**PCl (per ml/min)**	1.00	1-1.01	0.15	1.00	0.99-1.01	0.70	1.00	0.99-1.01	0.63	1.00	1-1.01	0.50
**Plasma Albumin (per g/l)**	0.92	0.88-0.97	<0.01	0.90	0.85-0.95	<0.001	0.90	0.85-0.95	<0.001	0.90	0.86-0.95	<0.001
**diabetes**	2.55	1.66-3.93	<0.001									
**IHD**				1.35	0.81-2.26	0.26						
**LVD**							1.62	0.9-2.91	0.11			
**PVD**										1.90	1.15-3.16	<0.05

DM, diabetes; IHD, ischemic heart disease; LVD, left ventricular dysfunction; PVD, peripheral vascular disease; PPCl, protein clearance; PSTR, peritoneal solute transport rate; AR, appearance rate; IL-6, interleukin-6

Supplementary Table 4: Predictors of Daily Peritoneal Protein Loss

	β	95%CI	P value
**PSTR (for each 0.1 increase)**	0.83	0.56-1.1	<0.001
**lg Dialysate IL6 AR (for each unit increase)**	0.552	0.01-1.09	<0.05
**lg Plasma IL6 (for each unit increase)**	-0.123	-0.42-0.17	0.41
**Plasma albumin (for each 1g/L increase)**	-0.006	-0.08-0.07	0.87
**Age (year)**	0.009	-0.01-0.03	0.41
**gender (compare with female)**	0.008	-0.62-0.63	0.98
**Comorbidity Grade 1 (compared with Grade 0)**	0.557	-0.13-1.24	
**Comorbidity Grade 2 (compared with Grade 0)**	0.672	-0.7-2.05	0.51
